# Prevalence and characteristics of metabolic dysfunction-associated steatohepatitis among pediatric patients in the MarketScan Databases

**DOI:** 10.1371/journal.pone.0334971

**Published:** 2025-10-27

**Authors:** Kennedy M. Peter-Marske, Xiao Zhang, Tongtong Wang, Xinyue Liu, Gail Fernandes, Samuel S. Engel, Ravi Shankar

**Affiliations:** 1 Merck & Co., Inc., Rahway, New Jersey, United States of America; 2 University of North Carolina at Chapel Hill, Chapel Hill, North Carolina, United States of America,; 3 Amgen, Thousand Oaks, California, United States of America; University of Benghazi, LIBYA

## Abstract

**Introduction:**

There are few epidemiologic studies of the prevalence of metabolic dysfunction-associated steatohepatitis (MASH) among pediatric populations.

**Objective:**

To estimate the cross-sectional prevalence of MASH in the pediatric (<18 years) populations of Merative^TM^ MarketScan® Commercial Database (Commercial) and Merative^TM^ MarketScan® Multi-State Medicaid Database (Medicaid).

**Methods:**

Pediatric patients with ≥1 medical encounter from 1/1/2020 to 12/31/2020 and ≥6 months of continuous enrollment before the most recent medical encounter were included. MASH was confirmed by an ICD-10-CM diagnosis code for MASH (K75.81) in the medical history since 1/1/2016.

**Results:**

A total of 1,476 and 410 pediatric MASH patients were identified in Medicaid and Commercial databases respectively, with a prevalence of 0.036% (95% confidence interval (CI): 0.034%, 0.038%) in Medicaid, as compared to 0.011% (95% CI: 0.010%, 0.012%) in Commercial. Prevalence of MASH increased by age. MASH was more prevalent in those who were male, obese, had T2D, and had metabolic syndrome. Among all pediatric MASH patients, the majority were ≥10 years old, male, and obese.

**Discussion:**

MASH is more prevalent among pediatric patients with comorbid conditions such as metabolic syndrome, obesity, and T2D, which appears to be similar to young adults. Rising prevalence of childhood obesity and related comorbid conditions, and the progressive nature of MASH, make this an area of increasing medical need. Prescreening MASH among high-risk patients with these comorbidities is needed for target intervention.

## 1. Introduction

Metabolic dysfunction-associated steatotic liver disease (MASLD) describes a range of disease states characterized by increased fat accumulation in hepatocytes. MASLD is predominantly the result of obesity-related insulin resistance, metabolic syndrome, type 2 diabetes (T2D), and various environmental and genetic factors [1–3]. Metabolic dysfunction-associated steatohepatitis (MASH), a progressive form of MASLD, consists of steatosis, inflammation, and varying degrees of fibrosis, which may progress further to cirrhosis and liver failure [1,4,5].

Currently, the global prevalence of MASH is estimated to be 5.27%, and the prevalent MASH cases will increase 63% from 16.52 million in 2015 to 27.00 million in 2030 [6,7]. As MASH is the most quickly increasing indication for liver transplant [8–10], the projected increase in prevalence makes this area a growing unmet medical need. MASLD is the most common form of pediatric liver disease and is associated with similar clinical risk factors as in adults [11,12]. While the entire spectrum of MASLD, including MASH, can occur among pediatric population, most existing reports of pediatric patient characteristics and prevalence are on MASLD instead of MASH. The prevalence of pediatric MASLD is estimated to be between 3% and 12%, but may be as high as 85% among pediatric patients with obesity [13–15]; prior reports suggest that the prevalence of MASLD increased by 26% among adolescents ages 15–19 over the past three decades [15]. Previous studies also report that pediatric MASLD is most prevalent among older, male, and Hispanic pediatric patients, as well as those with obesity, diabetes, obstructive sleep apnea, and panhypopituitarism [11]. Due to the need for a liver biopsy to definitively diagnose MASH [16,17], data on pediatric MASH patients are limited to single-center, tertiary care settings, or multicenter specialty networks [18–20].

MASH may progress and lead to greater risk for morbidity and liver-related mortality [4]. Additionally, MASLD and MASH in childhood may also be more severe than the same conditions among adults, and are both associated with worse cardiometabolic health measures and increased cardiac dysfunction in childhood [11,21]. Even though pediatric MASH is associated with serious adverse health outcomes, few pediatric patients are screened for MASH even when they exhibit comorbid conditions such as obesity, diabetes, and metabolic syndrome [22]. Since prior studies project an increase in MASH prevalence mirroring the rise in childhood obesity and obesity-related conditions [23], more research on MASH prevalence and patient characteristics is warranted.

To our knowledge, no published studies have reported the prevalence of MASH among pediatric populations overall and within different subgroups in the United States. A better understanding of the prevalence of MASH and characteristics of MASH pediatric patients may help inform future targeted interventions. Therefore, we conducted a cross-sectional study to assess the prevalence of MASH overall and by subgroups defined by patient demographics and comorbidity conditions, as well as characteristics of MASH patients, among the pediatric population in two separate United States administrative claims databases: Merative^TM^ MarketScan® Commercial Database (Commercial) and Merative^TM^ MarketScan® Multi-State Medicaid Database (Medicaid).

## 2. Methods

### 2.1 Data sources and study population

This study was conducted among pediatric population (<18 years) using structured, de-identified secondary data from the Merative^TM^ MarketScan^®^ Commercial and Medicaid databases. These two databases were selected to increase generalizability and diversity of the study population by including pediatric populations that were privately insured and publicly insured. Details of the Commercial and Medicaid databases and their enrollee characteristics have been previously described [24,25]. We utilized data on inpatient and outpatient service dates, procedure codes, and diagnosis codes, along with enrollment records. We included all pediatric patients with at least one encounter from January 1, 2020 to December 31, 2020 (index period), and who had at least 6 months of continuous enrollment before the index date (most recent medical encounter), with a maximum allowable enrollment gap of 45 days. Because all data were de-identified, this study was exempt from review by Institutional Review Boards.

### 2.2 MASH definition

MASH patients were identified as the presence of an International Classification of Diseases, 10^th^ Revision (ICD-10-CM) diagnosis code of K75.81 in their medical history after January 1^st^, 2016.

### 2.3 Subgroups

Predefined subgroups for analyses included age (0- < 2 years, 2- < 10 years, 10- < 15 years, or 15- < 18 years), sex (male or female), and prevalent comorbid conditions (metabolic syndrome, obesity, and T2D). Age groupings were selected based on clinical relevance. Comorbid conditions were assessed using all available data prior to and including the index date during the study period (see S1 Table for relevant codes).

### 2.4 Patient’s characteristics

MASH patient age, sex, comorbid conditions, medication use, diagnostic procedures, and healthcare utilization were described. Procedures commonly associated with diagnosing MASH were identified using Current Procedural Technology (CPT) codes in the three months prior to and following first MASH diagnosis during the study period, among those who met enrollment criteria during this period. Relevant diagnostic procedures included liver biopsy, computerized tomography (CT) scan of the abdomen, ultrasound of the abdomen, magnetic resonance imaging (MRI) of the abdomen, vibration-controlled transient elastography (FibroScan) with controlled attenuation parameter (CAP), and transient elastography (see S1 Table for information on identification of these procedures). Healthcare utilization measures were assessed, allowing for 6 months of lookback prior to start of the prevalence period (July 1, 2015 to the index date). Use of antidiabetic agents by T2D status was assessed at the index date.

### 2.5 Exploratory analysis

We also conducted an exploratory analysis among young adults (18- < 25 years) and compared the results to pediatric patients (<18 years).

### 2.6 Statistical analysis

All analyses were performed separately in the Commercial and Medicaid databases. The overall and subgroup-specific prevalence of MASH and its 95% confidence interval (CI) were calculated. For descriptive analyses of patient characteristics, continuous variables were reported as the mean (standard deviation) and the median. Categorical variables were summarized as the number and proportion of the total study population, and by predefined subgroups. All analyses were performed in SAS version 9.4 (SAS Institute, Cary, NC, United States).

## 3. Results

### 3.1 MASH prevalence

We identified 3,756,746 and 4,122,087 pediatric patients in the Commercial and Medicaid databases respectively, 410 and 1476 of whom had a MASH diagnosis. MASH prevalence among pediatric population was 0.011% (95% CI: 0.010%, 0.012%) in the Commercial database and 0.036% (95% CI: 0.034%, 0.038%) in the Medicaid database (Table 1). In both databases, MASH was more prevalent among males compared to females, and the prevalence increased with greater age; compared to the overall pediatric population, MASH was more common among pediatric patients with metabolic syndrome, obesity, and T2D.

**Table 1:** Prevalence of pediatric MASH^a^ (age < 18), overall, by age, sex, and comorbidity condition, based on the Commercial^b^ and Medicaid^c^ databases.

	Commercial			Medicaid		
	Total (N^d^) ^*^	MASH (N)	Prevalence (95% CI^e^)	Total (N)	MASH (N)	Prevalence (95% CI)
**Overall**	3,756,746	410	0.011% (0.010%, 0.012%)	4,122,087	1,476	0.036% (0.034%, 0.038%)
By age group						
0 to <2 years	264,502	3	0.001% (0.000%, 0.003%)	375,171	0	0.000% (0.000%, 0.000%)
2 to <10 years	1,613,867	28	0.002% (0.001%, 0.003%)	1,932,291	97	0.005% (0.004%, 0.006%)
10 to <15 years	1,136,686	161	0.014% (0.012%, 0.017%)	1,187,180	748	0.063% (0.059%, 0.068%)
15 to <18 years	741,691	218	0.029% (0.026%, 0.034%)	627,445	631	0.101% (0.093%, 0.109%)
By sex						
Male	1,909,518	267	0.014% (0.012%, 0.016%)	2,098,403	987	0.047% (0.044%, 0.050%)
Female	1,847,228	143	0.008% (0.007%, 0.009%)	2,023,683	489	0.024% (0.022%, 0.026%)
By comorbid condition						
Metabolic syndrome	6,223	80	1.286% (1.021%, 1.597%)	11,753	418	3.557% (3.229%, 3.907%)
Obesity	308,118	325	0.105% (0.094%, 0.118%)	621,577	1,360	0.219% (0.207%, 0.231%)
Type 2 Diabetes	4,894	35	0.715% (0.499%, 0.993%)	10,653	153	1.436% (1.219%, 1.681%)

* Numbers may not add up to total for mutually exclusive groups because of missing values.

Abbreviations: ^a^MASH-metabolic dysfunction-associated steatohepatitis^; b^Commercial-MarketScan^®^ Commercial Database; ^c^Medicaid-MarketScan^®^ Multi-State Medicaid Database; ^d^N-number; ^e^CI-confidence interval.

### 3.2 MASH patient characteristics

In both databases, most MASH patients were 10 years or older (>90%) and were more commonly male (>65%) (Table 2). In the Commercial database, metabolic syndrome, obesity, and T2D were observed in 19.5%, 79.3% and 8.5% of the MASH population, as compared to 28.3%, 92.1% and 10.4% in the Medicaid database. Just as the prevalence of comorbidities was higher in the Medicaid database, healthcare service utilization measures were generally higher among pediatric MASH patients in the Medicaid database compared to those in the Commercial database.

**Table 2:** Characteristics of pediatric MASH^a^ (age < 18) patients, based on the Commercial^b^ and Medicaid^c^ databases.

	Commercial N = 410	Medicaid N = 1,476
Age; n^d^ (%)		
0 to <2 years	3 (0.73%)	0 (0.00%)
2 to <10 years	28 (6.83%)	97 (6.57%)
10 to <15 years	161 (39.27%)	748 (50.68%)
15 to <18 years	218 (53.17%)	631 (42.75%)
Females; n (%)	143 (34.88%)	489 (33.13%)
Comorbidities; n (%)		
Metabolic syndrome	80 (19.51%)	418 (28.32%)
Obesity	325 (79.27%)	1,360 (92.14%)
Type 2 Diabetes	35 (8.54%)	153 (10.37%)
Healthcare utilization: mean ± standard deviation, median		
Number of unique pharmacological classes	4.6 ± 3.02, 5.0	6.4 ± 3.36, 6.0
Number of inpatient encounters	0.4 ± 1.27, 0.0	0.4 ± 1.37, 0.0
Number of emergency encounters	1.7 ± 3.15, 1.0	3.6 ± 4.94, 2.0
Number of ambulatory encounters	60.4 ± 72.91, 35.0	94.7 ± 145.76, 57.0

Abbreviations: ^a^MASH-metabolic dysfunction-associated steatohepatitis^; b^Commercial-MarketScan^®^ Commercial Database; ^c^Medicaid-MarketScan^®^ Multi-State Medicaid Database; ^d^N-number.

As shown in Table 3, among 364 patients in the Commercial database and 1,212 patients in the Medicaid database who had continuous enrollment 3-month prior to and after MASH diagnosis, only 14 (3.9%) and 106 (8.8%) had liver biopsy; approximately half had non-biopsy procedures only, and more than 20% did not have any diagnostic procedures 3-month prior to and after first MASH diagnosis during the study period (who may have had procedures completed outside the specified time frame).

**Table 3:** Procedures received in the 3 months prior to and following first MASH^a^ diagnosis during the study period among pediatric MASH patients (age < 18), based on the Commercial^b^ and Medicaid^c^ databases.

	Commercial N = 364	Medicaid N = 1,212
Procedures; n^d^ (%)		
Liver biopsy only	14 (3.85%)	106 (8.75%)
Liver biopsy + ≥1 non-biopsy procedure	50 (13.74%)	248 (20.46%)
Non-biopsy procedures only	189 (51.92%)	601 (49.59%)
No procedures	111 (30.49%)	257 (21.20%)

Abbreviations: ^a^MASH-metabolic dysfunction-associated steatohepatitis^; b^Commercial-MarketScan^®^ Commercial Database; ^c^Medicaid-MarketScan^®^ Multi-State Medicaid Database; ^d^N-number.

Among all pediatric MASH patients, metformin was the most commonly used antidiabetic agent with 15.1% of the patients from the Commercial database and 18.8% of the patients from the Medicaid database using this drug (Table 4). Eight and a half percent and 10.4% of pediatric MASH patients had T2D in the Commercial and the Medicaid databases respectively. Approximately similar proportions of patients in the two databases were on metformin without a concomitant diagnosis of T2D: 11.2% of patients in the Commercial database and 12.8% of patients in the Medicaid database respectively.

**Table 4:** Current antidiabetic agent use among pediatric MASH^a^ patients (age < 18), by T2D^b^ status, based on the Commercial^b^ and Medicaid^c^ databases.

	Commercial			Medicaid		
	Overall N = 410	T2D N = 35	No T2D N = 375	Overall N = 1476	T2D N = 153	No T2D N = 1,323
Antidiabetic agents*; n^e^ (%)						
DPP-4i^f^	2 (0.49%)	1 (2.86%)	1 (0.27%)	1 (0.07%)	1 (0.67%)	0 (0.00%)
GLP-1RA^g^	5 (1.22%)	4 (11.43%)	1 (0.27%)	19 (1.29%)	9 (6.04%)	10 (0.75%)
Insulin	13 (3.17%)	6 (17.14%)	7 (1.87%)	77 (5.22%)	28 (18.79%)	49 (3.69%)
Metformin	62 (15.12%)	20 (57.14%)	42 (11.20%)	278 (18.83%)	108 (72.48%)	170 (12.81%)
SGLT-2i^h^	1 (0.24%)	1 (2.86%)	0 (0.00%)	4 (0.27%)	3 (2.01%)	1 (0.08%)
Sulfonylurea	3 (0.73%)	1 (2.86%)	2 (0.53%)	5 (0.34%)	2 (1.34%)	3 (0.23%)
TZD^i^	3 (0.73%)	2 (5.71%)	1 (0.27%)	2 (0.14%)	1 (0.67%)	1 (0.08%)

*Patients may be using more than one anti-diabetic agent at once. Rows are not mutually exclusive.

Abbreviations: ^a^MASH-metabolic dysfunction-associated steatohepatitis; ^b^T2D-type 2 diabetes; ^c^Commercial-MarketScan^®^ Commercial Database; ^d^Medicaid-MarketScan^®^ Multi-State Medicaid Database; ^e^n-number; ^f^DPP-4i-dipeptidyl peptidase 4 inhibitor; ^g^GLP-1RA-glucagon-like peptide 1 receptor agonist; ^h^SGLT-2i- sodium-glucose cotransporter-2 inhibitor; ^i^TZD-thiazolidinediones

### 3.3 Exploratory analysis

Prevalence of MASH and characteristics of MASH patients among young adults age 18- < 25 were presented in S2 and S3 Tables. Overall, the prevalence of MASH was higher in young adults; the distribution of co-morbidities in young adults with MASH was similar to that in pediatric patients.

## 4. Discussion

In this study, an overall MASH prevalence of 0.011% and 0.036% was observed in the pediatric populations based on the Commercial and Medicaid databases, respectively. MASH was more prevalent among older subgroups, males, and patients with comorbidities such as metabolic disease, obesity, and T2D. Overall, MASH prevalence was higher among the pediatric patients from the Medicaid database than among the pediatric patients from the Commercial database.

Previous autopsy studies of pediatric population who died from conditions unrelated to MASH in San Diego and New York City found that 3% and 1.7% of the autopsy subjects had MASH. [26,27] Estimates from these autopsy studies are higher than the prevalence estimates observed in our study likely due to the underlying difference in overall health condition from the source populations. Additionally, liver histology was examined for all subjects in the autopsy studies and established histologic criteria (gold standard) were used to diagnose MASH, while in claims data collected for administrative purpose, cases may be identified using non-invasive procedures and there could be miscoding in diagnosis. Given the predominantly asymptomatic nature of the early stages of the disease, MASH is usually underdiagnosed in clinical practice. The aforementioned potentially led to an underestimation of the true prevalence of MASH in pediatric patients in this study.

Prior studies of pediatric MASH in the United States have predominantly consisted of patients with MASLD, and report the proportion of MASLD patients with confirmed MASH. However, these studies have not reported the prevalence of MASH among the general pediatric population, regardless of MASLD diagnosis. These studies, with sample sizes ranging from 87 to 780 pediatric patients with MASLD, report that 8% to 54% of pediatric patients with MASLD have “confirmed MASH”, while another 35% to 48% have “borderline MASH”, as determined by aggregate presence and degree of individual features of MASLD based on biopsy results [18–20,28–30].

Increased insulin resistance and changes in fatty acid and lipoprotein metabolism may link MASH pathogenesis with obesity, T2Ds, and metabolic syndrome [1,23]. As obesity and obesity-related conditions, along with MASH prevalence projections, have increased in pediatric populations, pre-screening for MASH among these high-risk subgroups may be particularly important [23]. However, few studies have investigated the prevalence of pediatric MASH in subgroups defined by comorbid conditions. In line with our results, others reported that subjects with MASH were more commonly older (average age of 17–18), males, and had a higher BMI. [26,27] A previous study that examined MASH in pediatric patients with T2D using United States healthcare claims data found the prevalence to be 0.6%, similar to the prevalence found among patients with T2D in the Commercial database in this study. [31] The slightly higher prevalence in the Medicaid database was likely due to enrollees characterized by low income and low social economic status, two social factors that are associated with higher risk of obesity and T2D which are important risk factors for MASH [32,33]. The potential discrepancy in race/ethnicity distribution between the two databases might also contribute to the observed difference, given the racial/ethnic disparity in MASH disease burden [34]. However, due to the incomplete information available on race/ethnicity in the claims databases, we were not able to verify our speculation. Additionally, we noticed that higher prevalence of MASH was observed in young adults age 18- < 25 than in pediatric population, and the distribution of comorbidities in young adults age 18- < 25 appeared to be similar to that in pediatric patients. One previous reported a high burden of steatosis among this age group, with prevalence of steatosis levels (S1-S3 Tables) among a population-based sample of United Kingdom young adults of 7.5% (S1 Table), 3.2% (S2 Table), and 10.0% (S3 Table) [35].

This study also characterized healthcare utilization, diagnostic procedures, and medication use among MASH pediatric patients. We found that though most pediatric MASH patients underwent a MASH-related procedure in the 3 months prior to or following first date of MASH diagnosis, very few underwent a liver biopsy. This indicates that while liver biopsy is the gold standard to diagnose MASH, due to its invasive nature, physicians may use other non-invasive tests instead in the pediatric population. This may also be true in the adult population, as one study reported that two thirds of MASLD patients do not undergo liver biopsy [36]. Since the agreement between non-invasive tests (NITs) and liver biopsy findings is less than perfect, it is possible that MASH is over-diagnosed in a patient with MASLD if only NITs are used. Whether non-invasive procedures or lab values can be reliably used to diagnose MASH in place of the gold standard of a liver biopsy in the pediatric population warrants further investigation.

With regard to medication use, we found that among pediatric patients carrying a diagnosis of MASH, metformin was the most commonly prescribed antidiabetic agent with 15% to 19% of MASH patients taking metformin. While patients with MASH and T2D were more likely to be prescribed antidiabetic agents, some patients were prescribed metformin even in the absence of a diagnosis of T2D. As shown in the study, 15.1% and 18.8% MASH patients were on metformin based on the Commercial and Medicaid databases, while only 8.5% and 10.4% MASH patients were diagnosed with T2D. Off-label use of antidiabetic agents among MASH patients without T2D may be for achievement of weight loss in MASH patients [37], which is consistent with the previous report that only 33% of pediatric metformin use was for T2D treatment [38].

### 4.1 Strengths and limitations

Our study used two healthcare claims databases with large sample sizes to estimate the prevalence of pediatric MASH among two populations with different enrollee makeups. The results of this study are largely generalizable to American pediatric population who are commercially insured and low-income pediatric populations who are supported by Medicaid. However, limitations should be noted. First, results are not representative of uninsured pediatric populations. Second, this study relied on administrative claims data and diagnosis codes collected for billing and record keeping purposes where clinical details and liver histology data are often missing. This limited our ability to assess the accuracy of MASH diagnosis, especially given the low biopsy rates for confirmation of diagnosis. We were also unable to investigate different lab based measures since the data were collected for administrative purposes, and lab values were largely missing. Additionally, use of the first MASH diagnosis code in available claims data may not necessarily reflect the true date of diagnosis, but rather the first encounter in which MASH was observed in that particular healthcare system. Due to this fact, reporting of diagnostic procedures surrounding first diagnosis date may be incomplete. Lastly, this study was limited by the realities of clinical practice. Though the study was able to document pediatric population who were diagnosed with MASH, we acknowledge that many pediatric patients may have remained undiagnosed. The lower prevalence of MASH patients with a diagnostic code versus prevalence estimated in the biopsy series shows potential underdiagnosis of MASH in real-world clinical practice.

## 5. Conclusions

MASH is more prevalent among pediatric patients with comorbid conditions such as metabolic syndrome, obesity, and T2D, which appears to be similar to young adults. Rising prevalence of childhood obesity and related comorbid conditions, and the progressive nature of MASH, make this an area of increasing medical need. Prescreening for MASH using non-invasive diagnostic measures and lab values among high-risk patients with these comorbidities is needed for target intervention.

## Supporting information

S1 Table Current Procedural Technology (CPT) codes and International Classification of Disease (ICD) codes used to define variables of interest. (DOCX)

S2 Table Prevalence of MASH^a^ (aged 18- < 25), overall, by age, sex, and comorbidity condition, based on the Commercial^b^ and Medicaid^c^ databases. (DOCX)

S3 Table Characteristics of pediatric MASH^a^ (aged 18- < 25), based on the Commercial^b^ and Medicaid^c^ databases. (DOCX)
