# Supplementary material for: Prevalence and characteristics of metabolic dysfunction-associated steatohepatitis among pediatric patients in the MarketScan Databases
Source: PLoS One. 2025 Oct 27;20(10):e0334971. doi: 10.1371/journal.pone.0334971 (PMC12558510; doi:10.1371/journal.pone.0334971)
Supplement: S1 Table — (DOCX) [file pone.0334971.s001.docx]

| **Supplemental Table 1**. Current Procedural Technology (CPT) codes and International Classification of Disease (ICD) codes used to define variables of interest | |
| --- | --- |
| Variable of interest | Codes |
| Computerized tomography (CT) scan of the abdomen | CPT codes: 74150, 74160, 74170, 74176, 74177, 74178 |
| FibroScan with controlled attenuation parameter (CAP) | CPT codes: 91200, 0346T |
| Liver biopsy | CPT codes: 47001, 47000, 47100 |
| Magnetic resonance imaging (MRI) of the abdomen | CPT codes: 74181, 74182, 74183, 74185 |
| Metabolic syndrome | ICD-10-CM code: E88.81  ICD-9-CM code: 277.7x |
| Obesity | CPT codes: 43644, 43645, 43647, 43648, 43770, 43771, 43772, 43773, 43774, 43842, 43845, 43846, 43881, 43882  ICD-10-CM codes: E66.0, E66.1, E66.2, E66.8, E66.9, Z68.54 |
| Transient elastography | CPT codes: 91200, 0346T |
| Type 2 diabetes | ICD-10-CM code: E11.x |
| Ultrasound of the abdomen | CPT codes: 93976, 76700, 76705, 93975, 76770, 76775 |
