# Supplementary material for: Prevalence and characteristics of metabolic dysfunction-associated steatohepatitis among pediatric patients in the MarketScan Databases
Source: PLoS One. 2025 Oct 27;20(10):e0334971. doi: 10.1371/journal.pone.0334971 (PMC12558510; doi:10.1371/journal.pone.0334971)
Supplement: S2 Table — (DOCX) [file pone.0334971.s002.docx]

| **Supplemental Table 2.** Prevalence of MASH^a^ (aged 18-<25), overall, by age, sex, and comorbidity condition, based on the Commercial^b^ and Medicaid^c^ databases | | | | | | | |  |  |  |  |  |  |
| --- | --- | --- | --- | --- | --- | --- | --- | --- | --- | --- | --- | --- | --- |
|  | **Commercial** | | |  | **Medicaid** | | |  |  |  |  |  |  |
|  | Total (N^d^) ^*^ | MASH (N) | Prevalence (95% CI^e^) |  | Total (N) ^*^ | MASH (N) | Prevalence (95% CI) |  |  |  |  |  |  |
| **Overall** | 1,768,454 | 1,003 | 0.057% (0.053%, 0.060%) |  | 772,619 | 920 | 0.119% (0.112%, 0.127%) |  |  |  |  |  |  |
| By age group | | | | | | | |  |  |  |  |  |  |
| 18 to <22 years | 995,925 | 513 | 0.052% (0.047%, 0.056%) |  | 535,457 | 643 | 0.120% (0.111%, 0.130%) |  |  |  |  |  |  |
| 22 to <25 years | 772,529 | 490 | 0.063% (0.058%, 0.069%) |  | 237,162 | 277 | 0.117% (0.103%, 0.131%) |  |  |  |  |  |  |
| By sex | | | | | | | |  |  |  |  |  |  |
| Male | 797,373 | 575 | 0.072% (0.066%, 0.078%) |  | 281,830 | 496 | 0.176% (0.161%, 0.192%) |  |  |  |  |  |  |
| Female | 971,081 | 428 | 0.044% (0.040%, 0.048%) |  | 490,788 | 424 | 0.086% (0.078%, 0.095%) |  |  |  |  |  |  |
| By comorbidity condition | | | | | | | |  |  |  |  |  |  |
| Metabolic syndrome | 9,898 | 146 | 1.475% (1.247%, 1.732%) |  | 7,441 | 204 | 2.742% (2.382%, 3.318%) |  |  |  |  |  |  |
| Obesity | 196,846 | 681 | 0.346% (0.320%, 0.373%) |  | 164,169 | 755 | 0.460% (0.428%, 0.494%) |  |  |  |  |  |  |
| Type 2 Diabetes | 16,578 | 149 | 0.899% (0.761%, 1.054%) |  | 12,774 | 213 | 1.667% (1.453%, 1.905%) |  |  |  |  |  |  |
| ^*^ Numbers may not add up to total for mutually exclusive groups because of missing values. | | | | | | | |  |  |  |  |  |  |
| Abbreviations: ^a^MASH-metabolic dysfunction-associated steatohepatitis; ^b^Commercial-MarketScan^®^ Commercial Database; ^c^Medicaid-MarketScan^®^ Multi-State Medicaid Database; ^d^N-number; ^e^CI-confidence interval. | | | | | | | |  |  |  |  |  |  |
