# Supplementary material for: Prevalence and characteristics of metabolic dysfunction-associated steatohepatitis among pediatric patients in the MarketScan Databases
Source: PLoS One. 2025 Oct 27;20(10):e0334971. doi: 10.1371/journal.pone.0334971 (PMC12558510; doi:10.1371/journal.pone.0334971)
Supplement: S3 Table — (DOCX) [file pone.0334971.s003.docx]

| **Supplemental Table 3.** Characteristics of pediatric MASH^a^ (aged 18-<25), based on the Commercial^b^ and Medicaid^c^ databases | | |
| --- | --- | --- |
|  | **Commercial**  N=1,003 | **Medicaid**  N=920 |
| Age; n^d^ (%) |  |  |
| 18 to <22 years | 513 (51.15%) | 643 (69.89%) |
| 22 to <25 years | 490 (48.85%) | 277 (30.11%) |
| Females; n (%) | 428 (42.67%) | 424 (46.09%) |
| Comorbidities; n (%) |  |  |
| Metabolic syndrome | 146 (14.56%) | 204 (22.17%) |
| Obesity | 681 (67.90%) | 755 (82.07%) |
| Type 2 Diabetes | 149 (14.86%) | 213 (23.15%) |
| Healthcare utilization; mean ± standard deviation, median | |  |
| Number of unique pharmacological classes | 5.8 ± 3.31, 6.0 | 8.0 ± 3.81, 8.0 |
| Number of inpatient encounters | 0.7 ± 2.27, 0.0 | 1.2 ± 3.22, 0.0 |
| Number of emergency encounters | 2.6 ± 5.27, 1.0 | 8.0 ± 12.88, 4.0 |
| Number of ambulatory encounters | 60.4 ± 71.61, 39.0 | 153.1 ± 252.67, 73.0 |
| Abbreviations: ^a^MASH-metabolic dysfunction-associated steatohepatitis; ^b^Commercial-MarketScan^®^ Commercial Database; ^c^Medicaid-MarketScan^®^ Multi-State Medicaid Database; ^d^N-number. | | |
